# Advances in Malaria Diagnostic Methods in Resource-Limited Settings: A Systematic Review

**DOI:** 10.3390/tropicalmed9090190

**Published:** 2024-08-23

**Authors:** Akua K. Yalley, Joyous Ocran, Jacob E. Cobbinah, Evangeline Obodai, Isaac K. Yankson, Anna A. Kafintu-Kwashie, Gloria Amegatcher, Isaac Anim-Baidoo, Nicholas I. Nii-Trebi, Diana A. Prah

**Affiliations:** 1 Department of Medical Laboratory Sciences, School of Biomedical and Allied Health Sciences, University of Ghana, Korle Bu, Accra P.O. Box KB 143, Ghana; akyalley@ug.edu.gh (A.K.Y.); aakafintu-kwashie@ug.edu.gh (A.A.K.-K.); gamegatcher@ug.edu.gh (G.A.); ianim-baidoo@ug.edu.gh (I.A.-B.); 2 Department of Biomedical Sciences, School of Allied Health Sciences, University of Cape Coast, PMB, Cape Coast, Ghana; joyous.ocran@stu.ucc.edu.gh (J.O.); jcobbinah@stu.ucc.edu.gh (J.E.C.); 3 Department of Virology, Noguchi Memorial Institute for Medical Research, University of Ghana, Accra P.O. Box LG 581, Ghana; eobodai@noguchi.ug.edu.gh; 4 CSIR-Building and Road Research Institute, Kumasi P.O. Box UP40, Kumasi, Ghana; ikyankson@csir.brri.org; 5 West African Centre for Cell Biology of Infectious Pathogens, University of Ghana, Legon, Accra P.O. Box LG 54, Ghana; 6 Department of Science Laboratory Technology, Faculty of Applied Sciences, Accra Technical University, Barnes Road, Accra P.O. Box GP 561, Ghana

**Keywords:** malaria, polymerase chain reaction (PCR), loop-mediated isothermal amplification (LAMP), diagnostic methods

## Abstract

Malaria continues to pose a health challenge globally, and its elimination has remained a major topic of public health discussions. A key factor in eliminating malaria is the early and accurate detection of the parasite, especially in asymptomatic individuals, and so the importance of enhanced diagnostic methods cannot be overemphasized. This paper reviewed the advances in malaria diagnostic tools and detection methods over recent years. The use of these advanced diagnostics in lower and lower-middle-income countries as compared to advanced economies has been highlighted. Scientific databases such as Google Scholar, PUBMED, and Multidisciplinary Digital Publishing Institute (MDPI), among others, were reviewed. The findings suggest important advancements in malaria detection, ranging from the use of rapid diagnostic tests (RDTs) and molecular-based technologies to advanced non-invasive detection methods and computerized technologies. Molecular tests, RDTs, and computerized tests were also seen to be in use in resource-limited settings. In all, only twenty-one out of a total of eighty (26%) low and lower-middle-income countries showed evidence of the use of modern malaria diagnostic methods. It is imperative for governments and other agencies to direct efforts toward malaria research to upscale progress towards malaria elimination globally, especially in endemic regions, which usually happen to be resource-limited regions.

## 1. Introduction

Malaria elimination has been a focal topic of public health discussions for the past decade or more. Despite being a tropically endemic parasitic infection, the impact of malaria is far reaching and remains a global health concern. The 2023 World Health Organization (WHO) report states that malaria cases rose to an estimated 249 million in 2022, with an increase of 5 million more cases from the year 2021 [1]. Although relentless efforts are being made and strategies put in place, much more is required to free our globe of the parasitic infection, particularly in indigenous malaria-endemic countries such as a number of sub-Saharan African countries where most cases occur [2].

Central to eliminating malaria is early, accurate detection, quantification, and differentiation of the parasitic infection, especially among asymptomatic persons. Asymptomatic plasmodium-infected individuals represent a major threat to malaria elimination worldwide as they do not show signs of clinical disease yet serve as parasite reservoirs and significantly contribute to the spread of the infection [3]. Notably, the majority of these asymptomatic infections are missed by conventional diagnostic techniques. As a result, the need for reliable, sensitive, and specific diagnostic or detection methods arises, which would also be useful for monitoring any decline in malaria transmission [4].

Technologies for malaria diagnosis have advanced in recent years; however, certain factors, such as the lack of laboratory infrastructure, operational costs, electricity requirements, and special operation expertise, have impeded the implementation of these advanced techniques in the vast majority of malaria endemic areas. This is especially the case when it comes to molecular testing, as these tests can be particularly expensive in addition to other challenges not only for malaria but for other infectious diseases [5]. The WHO describes microscopy (thin and thick film) as the primary method of detection [6]. Though microscopy is extensively used, it is unable to adequately detect low parasitemia, which is essential for effective treatment and subsequent elimination of the parasitic infection [7]. In addition, it is a laborious process requiring much expertise and experience for accurate diagnosis [4,8]. Other concerns have been the invasive approach of this technique, where blood samples are collected after a painful pierce of a needle, and yet an accurate diagnosis unassuredly relies solely on the discretion of the laboratory scientist. In several developing countries, there is inadequate expertise, equipment, and supplies required for accurate detection; as such, there are greater risks of contamination and false diagnosis [9]. Furthermore, it becomes more unreliable and difficult to distinguish low level infections as transmissions decline; hence, there is a need for alternative approaches to detection as elimination is being considered [4].

Can there be a faster, more specific, and more sensitive method of detecting malaria that can easily be implemented in resource-limited areas? The question remains among scientists globally. Can malaria be eliminated and many more lives saved by the emergence of technologies that offer early detection and differentiation of very minimal malarial infections? Does mankind stand a chance of advancement towards needle-free malaria detection, point-of-care devices, and personalized malaria medicine? For lower and lower-middle-income countries, which total 26 and 54, respectively (Table 1), according to the World Bank, will there be access to such effective diagnostic tools [10]? It is worthy of note that 11 of these countries, all in sub-Saharan Africa, bear 70% of the global malaria burden, according to the 2023 WHO report [1] (Table 1). The above-mentioned questions are but a few that remain on the minds of scientists and thus drive research.

Subsequently, there are several techniques that have been developed over the years to address some of the challenges with the gold standard technique. Rapid diagnostic tests (RDTs) are fast and reliable. Malaria RDTs do not require skilled personnel or constant electricity, but relative to malaria microscopy, they are expensive, have a short shelf life, and only give qualitative results [11]. Other diagnostic techniques, such as enzyme-linked immunosorbent assay (ELISA), lateral flow immunoassay (LFIA), microarrays, aptamer-based biosensors, genomic sequencing, loop-mediated isothermal amplification (LAMP), nested PCR, real-time PCR, and quantitative nucleic sequence-based amplification, are usually reserved for research and surveillance purposes. These techniques have higher sensitivity and specificity for malaria diagnosis relative to microscopy and RDTs. Notwithstanding, some of them are more laborious and expensive to deploy in resource-limited areas.

This systematic review looks at traditional and modern techniques in light of their main advantages and disadvantages, as well as the countries where they have been used, with emphasis placed on lower and lower-middle-income countries. Also, emphasis would be placed on molecular-based techniques and how common they are in resource-limited settings, which usually happen to be endemic to malaria.

## 2. Materials and Methods

In conducting this systematic review, an accurate and authentic outcome was ensured by adherence to the Preferred Reporting Items for Systematic Reviews and Meta-Analysis (PRISMA) guidelines. The review was registered in the open science framework database (https://doi.org/10.17605/OSF.IO/DV6Z3 (accessed on 28 June 2024)). Relevant details needed were obtained from articles published in journals and from databases up to 2022 since the search was done in early 2023. Key search phrases used for the search included “malaria detection methods”, “emerging technologies in malaria”, “recent advances in malaria detection or diagnosis”, “emerging methods in malaria diagnosis”, “traditional methods of malaria detection”, “Point of care devices for malaria detection”, “Non-invasive or needle-free malaria detection”, and “personalized malaria medicine”. Numerous articles were obtained from databases, journals, and other publishing sites, including Google Scholar, PUBMED, and MDPI databases.

From the databases, a total of 327 were identified. After the searches, the publications were sorted out to remove duplicates, and 20 publications were removed. The records were further screened to remove all incomplete, unpublished articles, and ineligible publications. With articles published in recent years under consideration, all accessible publications were considerable options, leaving out articles from journals that needed to be purchased, were restricted, or there was not a PDF version of the complete paper readily available.

Upon abstract screening, articles were selected based on the following general criteria: a traditional or modern method of malaria detection or diagnosis was investigated. A total of 276 articles were obtained, uploaded into Mendeley Reference Manager and Endnote, and carefully reviewed for full text eligibility and results presentation.

**Figure 1:**
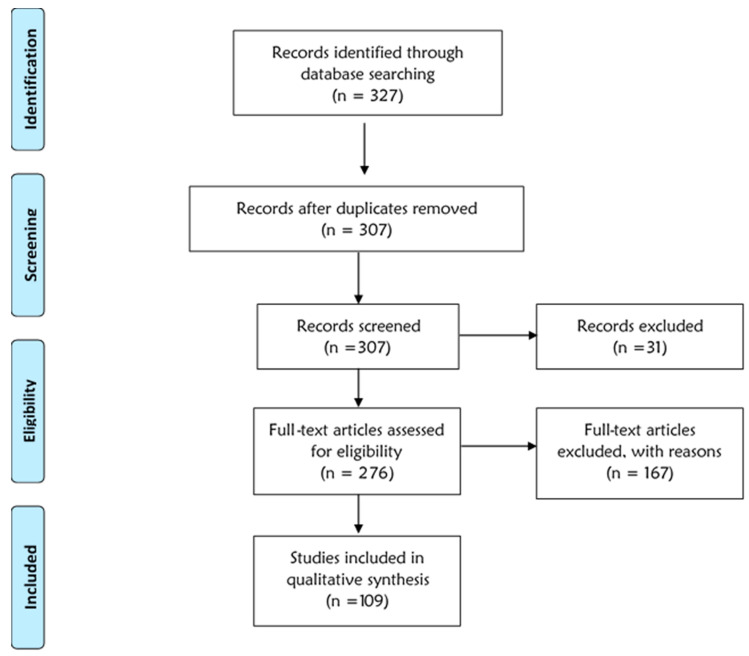
A PRISMA flow diagram showing the method of article selection.

Of these articles, some investigated traditional methods, while most investigated various modern methods. Some articles were also found useful as they investigated or reviewed “emerging technologies in malaria detection” or “advances in malaria detection”, among others, and thus were used in the other parts of the review writing. After a comprehensive document screening, 167 were later removed as it was found that they did not suit the review criteria, leaving 109 to be reviewed. These articles were removed for reasons including the papers being published earlier than 2014 (for those to be analyzed), they did not specifically investigate diagnostic tools for malaria, the article obtained was not the published version, and the research scope and contents were not clear or did not focus on a possible malaria detection method. In several instances, multiple malaria detection methods were identified in a single publication; thus, the number of developed methods identified exceeded the number of publications used. Figure 1 below shows the sorting-out process.

## 3. Results

### 3.1. Traditional Methods Used for Malaria Detection

Table 2 below shows a summary of traditionally used methods of malaria detection, elaborating on their approach as well as the pros and cons of using these methods for diagnosis. Though there has been the development of new and innovative methods of detection over the years, microscopy, using thick and thin blood films coupled with Giemsa staining, remains the gold standard for the diagnosis of malaria parasitic infections [8,12].

### 3.2. Modern Methods Used for Malaria Detection

The quest to effectively treat malaria while gravitating towards its elimination has driven the development of various tools and assays for the diagnosis of malaria (4). Table 3 and Table 4 contain recently developed methods used in the diagnosis of malaria and where they have been used. These diagnostic approaches vary greatly, ranging from biosensors and molecular assays down to computerized algorithms and automated analyzers, which have been developed or used over recent years, no earlier than 2014. The advantages and limitations of each diagnostic method are considered, as well as the summarized procedure by which it is conducted.

Table 5 analyzes evidence of the use of some recently developed detection tools in lower and lower-middle-income countries where there are often resource limitations. The test types that featured most frequently in publications were PCR techniques (eleven), followed by RDT tests (nine), then LAMP techniques and computerized/digital deep machine learning approaches (six each). In all, twenty-one countries had publications featuring modern malaria diagnostic methods.

**Figure 2:**
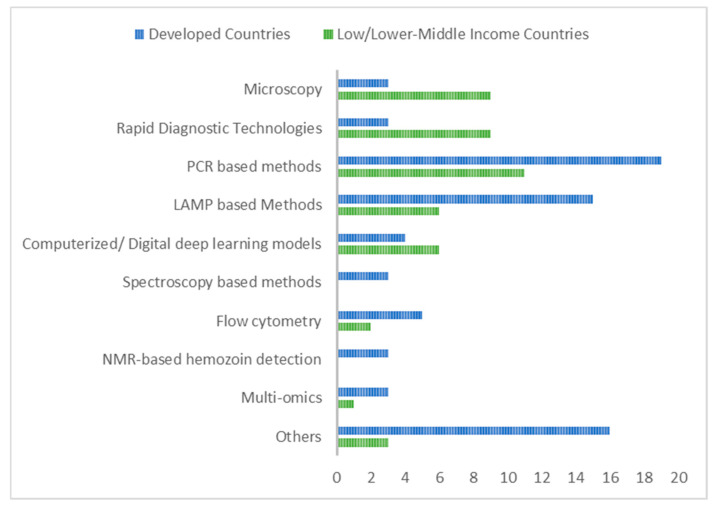
Frequencies of malaria detection/diagnosis methods in reviewed publications.

In Figure 2, the various methods of malaria detection reported from the identified studies have been represented graphically, indicating which diagnostic trends are being largely investigated, used more, or have gained much research interest. The chart represents malaria diagnostic developments investigated from 2014 until 2022. PCR-based methods and LAMP-based methods were the most prevalent methods.

## 4. Discussion

Critical to achieving effective control, treatment, and subsequent elimination of malaria is the timely detection of the parasitic infection. In the face of this threatening infection, continuous progress and innovative research are required, which leads to the development of new tools that will be useful in the fight against malaria [117]. This article reviewed the recent developments in malaria diagnostic methods and their potential for point-of-care and personalized malaria care, with special emphasis on the use of these methods in economically challenged countries.

The findings from this review suggest great advancement recently in malaria diagnostics. Research efforts by many scientists around the globe have progressed from developing improved malaria microscopy techniques into enhanced and more accurate molecular, immunological, computerized, digital methods of detection, automated analyzers, and point-of-care devices. Studies suggest that the influence of the old age infection on global health outcomes has urged on the design of more efficient diagnostics, with efforts directed at the development of point-of-care devices useful for resource-limited areas [7]. For an active drive towards the elimination of malaria, an early detection approach capable of revealing low levels of the parasitic infection is imperative [3].

As observed in Table 2, Table 3, Table 4 and Table 5, the outcome of this review indicates that recent malaria detection methods actively being used or investigated include traditional methods, molecular techniques with polymerase chain reaction (PCR), loop-mediated isothermal amplification (LAMP)-based assays, and machine learning/computerized techniques (that exploit the physical and/or biological properties of Plasmodium-infected erythrocytes to enhance malaria diagnosis), among others. Figure 2 shows the frequencies of detection methods as identified from various articles published over the last decade. Several other technologies and chemical assays are also being designed to tackle the malaria burden. RDTs were among the commonly used or researched modern methods in resource-limited settings, as seen in Table 5 and Figure 2. This is not surprising since they are relatively low cost and easy to use.

Studies confirm PCR-based techniques as having widespread use globally as they are highly sensitive and capable of detecting very low parasitemia levels [3,22,35]. Polymerase chain reaction basically makes use of DNA extracted from whole blood or other samples. The process continues with denaturation, amplification, and elongation steps, after which the sensitivity and specificity of the assay can be assessed [35]. It is proposed that the PCR method, under equal reaction parameters, can diagnose all five species of the plasmodium parasitic infection [35]. Our findings reveal that a wide range of PCR assays have been developed or used over the past decade, which are less laborious and provide much faster and more accurate results [22]. Furthermore, PCR-based assays are widely preferred due to several reasons, including simultaneous species-specific detection and quantification, higher sensitivity, higher specificity, less time consuming, easy to use, and capable of diagnosing subclinical infections [13,18,22,23,41,42].

Though PCR is an effective approach to malaria detection, it is limited by the requirement of costly laboratory facilities and expertise and thus less beneficial to resource-limited areas and at the point of care [3]. Despite that, quite a number of studies in resource-limited settings, including some African countries, utilized PCR-based techniques, as shown in Table 5 and Figure 2 [13,16,18,23,25,29,35,36,37,38,40]. Other advanced PCR techniques, such as lab chip real-time PCR (LRP) and hair qPCR, were found to be suitable alternatives for point-of-care or resource-limited settings, though no evidence was found of the former currently being used or researched in lower or lower-middle-income countries [29,31]. Gómez-Luque et al. proposed that due to limitations observed, more research is required to affirm the use of the hair qPCR as an efficient technique for malaria detection [29]. The one advantage the hair qPCR has over other PCR types is the use of non-invasive samples. LRP, however, being highly sensitive, specific, and less expensive will be beneficial for diagnosis and control in malaria-endemic countries [31].

LAMP-based assays have also dominated research on malaria diagnostics. As seen in Table 3, studies have shown the development or use of various LAMP assays, which are effective malaria diagnostics [118]. Rei Yan et al. reviewed LAMP assays and found them easy to use in regions where there is limited access to clinical expertise and molecular biology equipment. Modified LAMP based assays such as multiplex LAMP with dipstick DNA chromatography, high throughput LAMP, 18S rRNA LAMP, mediated LAMP combined with lateral flow detection (LFD), etc., are highly sensitive, easy to use, consistent, convenient, cost effective, and useful in point-of-care situations [55,56,58,59], thus enabling an approach towards personalized healthcare. Table 5 provides evidence of the development and use of LAMP techniques in lower and lower-middle-income countries, including countries in sub-Saharan Africa where malaria is endemic [45,48,56,57,58,59].

Other molecular methods worthy of note as they double as point-of-care or easy-to-use methods include nuclear magnetic resonance (NMR)-based hemozoin detection, ultra-bright SERS nanorattles, recombinase-aided amplification with lateral flow dipstick assay, and dye-coupled aptamer-captured enzyme-catalyzed assay [86,104,105,110,111,112]. Though the latter two could be used in resource-limited settings due to their low cost, the study found only a dye-coupled aptamer-captured enzyme-catalyzed assay used in India [104]. Veiga and Peng identified nuclear magnetic resonance (NMR)-based hemozoin detection as having the potential of enabling personalized malaria medicine (that is, malaria treatment tailored to individual characteristics) with needleless diagnosis foresighted [119]. This technology may offer the detection of phenotypic variants, which are observable variations in characteristics among parasites of the same species as a result of genetic diversity, host–parasite interactions, or environmental factors, among others [120,121]. For example, there are drug-resistant variants, those with surface antigen variations, and variants with different clinical presentations, among others [121,122,123]. The ability to detect such variants would increase diagnostic accuracy and be considerably useful against parasite drug resistance. Acquiring these time- and patient-specific phenotypic identifiers is a basic step to personalized malaria medicine as variants continually rise [119]. The one advantage that phenotypic variant determination using NMR technology may have over nucleic acid amplification-based methods for genomic profiling is the extremely fast turnaround time for some of the devices [110]. Unfortunately, there was no evidence of such methods being used in lower and lower-middle-income countries as per the studied published data in the research articles reviewed.

Furthermore, a number of other technologies have emerged capable of point-of-care diagnosis. Unlike the traditional microscopy and commonly used RDTs, some of these methods were found to be highly sensitive, non-invasive as far as sample collection was concerned, and cost effective, even though there was no evidence that cost-effective ones were necessarily being used in economically challenged settings [85,95,99,100,101,102]. In addition to these, Aggarwal et al. classify omics-based diagnostics as another important category to malaria diagnosis and elimination [124]. Multi-omics combines genomics, proteomics, metabolomics, phenomics, and transcriptomics in the investigation of biomarkers optimal for disease diagnosis and treatment. Though each omics has individual limitations, collectively, multi-omics can lead to a more comprehensive understanding of malaria infections, which can lead to more effective treatments [124]. In this review, the only struggling economy we found using multi-omics was India. No African nation was indicated.

## 5. Conclusions

Given the literature reviewed, there is adequate evidence to suggest that malaria detection or diagnosis will progress significantly in the next decade and beyond towards needleless detection. This advancement will however require increased, detailed, and specified research into the various molecular identifiers and phenotypic variant characteristics of malaria infection while enhancing the accuracy, precision, and specificity of the modernized point-of-care diagnostic tools. With this in view, precedence is duly set for the use of personalized medicine in the treatment of malaria infections. Notwithstanding, the traditional thin and thick film microscopy and RDTs will continue to play an important role in the accurate detection of malaria infections, especially in resource-limited areas where there is less access to modernized diagnostic tools and little research into advanced malaria detection methods. It is, however, encouraging to see that PCR-based and LAMP-based tests were seen being utilized in these areas, including African countries. However, other modern molecular/point-of-care tests were not being utilized in sub-Saharan Africa. Findings of this study show that approximately a quarter (26%) of a total of eighty countries in low and lower-middle-income settings employ state-of-the-art methods for malaria diagnostics. This underscores the need for governments, non-governmental organizations, and funding bodies to intensify efforts towards malaria diagnostics and research in the fight against malaria.

## Figures and Tables

**Table 1:** Countries classified by World Bank as low or lower-middle-income economies in 2024 [1,10]. The table lists all resource-limited countries divided into low (top portion) and lower-middle (bottom portion) income countries, with special emphasis placed on the 11 countries (right portion) that together bear 70% of the global malaria burden.

	LOW AND LOWER-MIDDLE INCOME COUNTRIES								
								70% GLOBAL MALARIA BURDEN	
LOW-INCOME COUNTRIES	Afghanistan	Burundi	Central African Republic	Chad	Eritrea	Ethiopia	Gambia	Burkina Faso	Congo, Dem. Rep
	Guinea-Bissau	Korea, Dem. People’s Rep	Liberia	Madagascar	Malawi	Rwanda	Sierra Leone	Mali	Mozambique
	Somalia	South Sudan	Sudan	Syrian Arab Republic	Togo	Yemen, Rep		Niger	Uganda
LOWER-MIDDLE INCOME COUNTRIES	Angola	Algeria	Bangladesh	Benin	Bhutan	Bolivia	Cabo Verde	Cameroon	Ghana
	Cambodia	Comoros	Congo, Rep.	Côte d’Ivoire	Djibouti	Egypt, Arab Rep.	Eswatini	India	Nigeria
	Guinea	Haiti	Honduras	Jordan	Iran, Islamic Rep	Kenya	Kiribati	Tanzania	
	Kyrgyz Republic	Lao PDR	Lebanon	Lesotho	Mauritania	Micronesia, Fed. Sts.	Mongolia		
	Morocco	Myanmar	Nepal	Nicaragua	Pakistan	Papua New Guinea	Philippines		
	Samoa	São Tomé and Principe	Senegal	Solomon Islands	Sri Lanka	Tajikistan	Timor-Leste		
	Tunisia	Ukraine	Uzbekistan	Vanuatu	Vietnam	Zambia	Zimbabwe		

**Table 2:** Traditional methods used for malaria detection.

Traditional Methods	Specimen Used	Summary of Procedure	Invasive/Non-Invasive	Advantages	Disadvantages	Refer-ences
Thin film microscopy	Blood	Thin blood smears are prepared and stained using Giemsa stain. Thin smears are examined with a 100× oil immersion objective.	Invasive	Reliable in the identification of four human plasmodium species and their various stages	Limited by quality of blood smears as well as availability of skilled microscopists. Lack of sensitivity where non-falciparum or mixed infections exist.	[8,13,14,15,16,17,18]
Thick film microscopy	Blood	Thick blood smears are prepared and stained using Giemsa stain. Thin smears are examined with a 100× oil immersion objective.	Invasive	Reliable in the detection of four human plasmodium species	Limited by quality of blood smears as well as availability of skilled microscopists.	[8,13,14,15,16,17,18]
Morphology-based diagnosis	Blood	Optical images from Giemsa-stained infected blood are measured using Olysia and Scanning Probe Image Processor software based on morphology of red blood cells.	Invasive	Faster prediction of malaria cases	Expertise needed	[19]
Centrifuged buffy coat smear examination (CBCS)	Blood	Centrifugation of buffy coat is done prior to Giemsa staining and microscopic examination	Invasive	Specificity is similar to conventional method but sensitivity a bit better than conventional method	Limited by availability of skilled microscopists	[20]

**Table 3:** Modern (PCR/LAMP-based) methods used for malaria detection and evidence of use in developed countries.

Modern Methods	Specimen Used	Description	Invasive/Non-Invasive	Point of Care/Molecular/Other	Advantages	Disadvantages	Developed Countries	References
Direct conventional PCR	Blood	With plasmodium cytochrome oxidase III gene (COX-III) as target, direct conventional PCR is conducted on bloodspot samples. Results are visualized on a gel.	Invasive	Molecular	High Sensitivity; faster than nested; does not require DNA isolation	Requires much expertise and expensive	USA	[21]
Nested Polymerase Chain Reaction (PCR)	Blood	Using different primer pairs to run 2 sequential amplification reactions. Plasmodium genomic DNA extracted from dried blood spots	Invasive	Molecular	High sensitivity and specificity	Time consuming, expensive, requires much expertise	Thailand, USA, Brazil, United Kingdom, Austria	[13,16,18,21,22,23,24,25]
Droplet Digital PCR (ddPCR)	Blood, Serum	DNA extracted from blood and serum samples are analyzed using the ddPCR method, which is based on water–oil emulsion droplet technology	Invasive	Molecular	High sensitivity using blood samples	Low sensitivity using serum samples; expensive	Italy, Thailand	[26,27]
Photo- Induced Electron transfer PCR (PET-PCR)	Blood	Total DNA is extracted from dried blood spots and PCR performed using photo-induced electron transfer fluorogenic primers	Invasive	Molecular	High sen-sitivity for parasite identification and characterization.	Requires much expertise and is expensive	USA	[15]
Fluoresen-ce reson-ance energy transfer (FRET) real time PCR	Blood	Real-time PCR utilizing FRET whereby fluorophores are brought in close proximity after hybridization is performed on DNA extracted from lyophilized blood samples targeting the 18S rRNA gene	Invasive	Molecular	High sensit-ivity, and allows for simultaneous quantitative and species-specific detection	This specific protocol could not differentiate between *P. vivax* and *P. knowlesi;* expensive	United Kingdom, Austria	[22]
SYBR Green Real-Time PCR-RFLP Assay	Blood	Real-time PCR using sybr green dye that binds to all double-stranded DNA followed by restriction fragment polymorphism to differentiate species	Invasive	Molecular	High sensitivity	Meltcurve required in PCR since Sybr green alone can be non-specific; expensive	Sweden	[28]
Hair qPCR	Head hairs	Hairs without roots are taken from patients and qPCR assay conducted	Non-invasive	molecular	Requires no special trans-port/storage conditions for samples	Sensitivity lower than when blood samples are used	Spain	[29]
Insulated Isothermal PCR (iiPCR)	Blood	PCR is performed in a portable device using an assay based on the Rayleigh–Bénard convection method	Invasive	Molecular/point of care	Portable, easy and fast operation; direct interpretation	Not as sensitive as qPCR	Malaysia	[30]
Lab Chip Real Time PCR (LRP)	Blood	DNA is extracted from collected blood samples and a portable LRP device is used to detect malarial parasites based on lab-on-chip technology	Invasive	Molecular/point of care	High sensitivity and specificity. Fast and cost effective	Risk of false negatives higher than traditional real-time PCR	Korea	[31]
Pv-mt Cox PCR	Blood	DNA is extracted from collected blood samples and qPCR with mitochondrial gene target is carried out	Invasive	Molecular	More sensitive in the detection of *P. vivax*	Expensive	Brazil	[32]
PvLAP5 and Pvs25qRT-PCR assays	Blood	Extracted RNA is subjected to quantitative reverse transcription PCR	Invasive	Molecular	Suitable assay for the determination of human transmission reservoir	Expensive	Panama	[33]
Other Quantita-tive PCR (qPCR)	Blood	Real-time PCR performed using primers targeting different regions and SYBR green or probe-based technology on DNA extracted from whole blood	Invasive	Molecular	High sensitivity and rapid	Extreme caution needed to prevent contamination; expensive	France, Canada, USA Columbia Germany, Brazil, China, Malaysia	[34,35,36,37,38,39,40,41,42,43,44]
Dry LAMP system (CZC-LAMP)	Blood	Blood samples are analyzed directly without extraction using the assay made up of dried reagents	Invasive	Point of care/molecular	High sensitivity and specificity; no need for prior extraction	Not widely available		[45]
Particle Diffusometry (PD)-LAMP	Blood	PD, which senses images, is combined with LAMP on a smartphone-enabled device to detect low levels of parasitemia	Invasive	Point of care/molecular	Sensitivitities higher than RDTs and similar to qPCR	Sensitivity slightly lower than nested PCR	USA	[46]
LAMP and MinION™ nanopore sequencer	Blood	Primers targeting the 18S–rRNA gene of all five human Plasmodium species are generated and samples subjected to LAMP. Min-ION™ nanopore sequencer is used on amplicons to identify *Plasmodium* spp.	Invasive	Molecular	Highly sensitive, and simple	Expensive	Japan	[47]
Other Loop-mediated isothermal amplification (LAMP),	Blood	Extracted DNA is subjected to loop-mediated isothermal amplification with a variety of detection methods	Invasive	Point of care/molecular	Simple, low cost; can be used in resource-limited settings and point-of-care settings	Some cannot quantify par-asite density; some are insensitive towards low parasitemia and mixed infections	France, Korea, Thailand Italy, Brazil Spain, Mala-ysia, Japan, Peru, USA	[26,34,48,49,50,51,52,53,54,55,56,57,58,59,60,61,62,63]

**Table 4:** Modern (non-PCR/non-LAMP-based) methods used for malaria detection and evidence of use in developed countries.

Modern Methods	Specimen Used	Description	Invasive/Non-Invasive	Point of Care/Molecular/Other	Advantages	Disadvantages	Developed Countries	References
Malaria SD Bioline RDT kit	Urine, Saliva, Blood	Using immuno-chromatography to detect PfhRP2 and PLDH following manufacturer’s instructions	Non-invasive/Invasive	Point of care	Effective for non-invasive detection of malaria; low cost	Low sensitivity		[64]
Other (RDTs)	Blood	Immunochromatography/ according to manufacturer’s instructions	Invasive	Point of care	Suitable for point of care in hard-to-access areas; low cost	Low sensit-ivity for some kits; poor identification of non-falciparum infections for some brands	Indonesia Australia, USA	[14,15,17,18,65,66,67,68,69,70,71]
Computeri-zed/digital deep mach-ine learnin-g approach	Blood	Machine learning models are used to detect malaria parasites in blood smears. Some can be integrated into smartphone detection apps	Invasive	Other	Accurate/ reliable	For some, results are affected by quality of smears	USA, Taiwan, China, Turkey	[72,73,74,75,76,77,78,79,80,81]
Spectros-copy	Blood	Blood samples are analyzed using spectroscopy	Invasive	Other	Highly effective for identifying infected cell	Only qualitative results obtained	Thailand, China, Australia	[82,83,84]
Portable Optical Diagnostic System (PODS)	Blood	Works by differential optical spectroscopy. The change in optical power before and after a magnet is applied, is monitored in order to determine β-hematin concentration in whole blood	Invasive	Point of care	Portable; low cost; useful for low resource settings; high sensitivity	Not widely available	USA	[85]
Ultra bright SERS nanorattles	Blood	DNA detection method that uses sandwich hybridization of magnetic bead, target sequence, and ultrabright SERS nanorattle are employed	Invasive	Molecular/point of care	Sensitive; can be automated and added to portable devi-ces for POC diagnosis; can identify SNPs hence, discri-minate betw-een wild-type and mutant parasites	Not widely available	USA	[86]
Automated Microscopy/Digital Analysis	Blood	Comprises a fluorescent dye staining or Giemsa staining and an automated microscopy platform and digital analysis	Invasive	Other	Rapid diagn-osis and par-asite density monitoring. High sens- itivity, linear-ity, and precision	Not widely available	Korea, Finland, Sweden	[87,88,89]
Flow cytometry	Blood	Parasites are detected and quantified in blood by use of analyzers utilizing flow cytometry technology	Invasive	Molecular	Rapid and high sensiti-vity; useful for mass screening	May not be able to distinguish plasmodium species	Netherlands, France, USA, South Africa, Japan	[90,91,92,93,94]
Thin-Film Optical Filters	Blood	A thin film optical device is used based on optical reflectance spectrophotometry, for the parasite detection through haemozoin quantification	Invasive	Point of care	High sensitivity	High transmittance regions outside target wavelength	Portugal	[95]
Rotating cr- ystal magn-eto optical detection (RMOD) method	Blood	RMOD works by detection of the periodic modulation of light transmission. This is induced by hemozoin crystals which co-rotates with a rotating magnetic field	Invasive	Other	Higher sensitivity and accuracy than light microscopy	Sensitivity is poorer than PCR methods	Thailand, Hungary	[96,97,98]
Hemozin-Based Malaria diagnostic device (Gazelle^TM^)	Blood	Using magneto-optical technology, the device detects hemozoin produced by Plasmodium	Invasive	Other	Sensitivities comparable to light micr-oscopy; faster than micros-copy; portab-le; can run on battery power	Unable to distinguish between species		[16]
Hemozoin-generated vapor nanobubbles	Blood vessel (transdermal)	Hemozoin generates a transient vapor nanobubble around hemozoin in response to a short and safe laser pulse. The acoustic signals of these nanobubbles that are malaria specific enable detection	Non-invasive	Point of care	Non-invasive; rapid	Not widely available	USA	[99]
Electroche-mical immunosensor	Blood	Egg yolk IgY antibodies against Plasmodium vivax lactate dehydrogenase antigen are immobilized on a gold electrode surface followed by differential pulse voltammetry and contact angle measurements are made.	Invasive	Point of care	High Sensitivity for malaria caused by *P. vivax*	Only malaria caused by *P. vivax* can be detected	Brazil	[100]
Simplified ELISA)/PfHRP 2 ELISA	Blood	Modified ElISA was performed on blood samples.	Invasive	Point of care	High sensitivity, portable and low cost	Not widely available	Spain UK Denmark	[101,102]
Multiple-xed ELISA based assay	Blood	Multiplexed ELISA-based (either planar-based array or magnetic bead-based platforms) technologies are used for malaria detection	Invasive	Molecular	Can detect malaria spe-cies mutants; have high throughput potential	Not widely available	USA	[103]
Dye-Cou-pledApt-amer-Capt-ured Enzy-me-Cataly-zed assay	Blood	Aptamer- and enzyme-based method is used to detect malaria infection in blood. Method can be used on instrument or instrument free platform	Invasive	Molecular/point of care	Low cost; useful for resource-limited and point-of-care settings.	Not widely available		[104]
Recombinase-Aided Amplificat-ion with Lateral Flow Dip-stick Assay (RAA-LFD)	Blood	A combination of recombinase-aided amplification lasting for 15 min at 37 degrees and lateral flow dipstick is used to detect plasmodium species in blood	Invasive	Molecular/point of care	Highly sensitive, specific, low cost, convenient for on-site screening and low resource settings.	Not widely available	China	[105]
Portable image-based Cytometer	Blood	*P. falciparum*-infected blood cells are identified and counted from Giemsa-stained smears using the image based portable cytometer.	Invasive	Other	Simple to operate; low cost	Not widely available	Singapore	[106]
Two-stage sample-to-answer sy-stem based on nucleic acid ampl-ification approach	Blood	It combines the dimethyl adipimidate (DMA)/thin film sample processing (DTS) technique and the Mach–Zehnder interferometer isothermal solid-phase DNA amplification (MZI-IDA) technique to detect infection in blood	Invasive	Molecular	High sensitivity, rapid	Not widely available	Singapore, Korea	[107]
Fluorescen-ce In Situ Hybridization (FISH) Assays	Blood	Detects and localizes specific malaria nucleic acid sequences by hybridizing with complementary sequences that are labeled with fluorescent probes	Invasive	Molecular	High sensitivity	Skilled expertise required.	USA	[108,109]
NMR-based hemozoin detection	Blood	Detection is based on the ability to recognize the paramagnetic susceptibility of malaria hemozoin crystals	Invasive	Molecular/point of care	High sensitivity and rapid	Not widely available	Australia, Singapore, USA	[110,111,112]
Multi-omics	Varies	Integrating data from different omic methods	Invasive/non-invasive	Other	Comprehen-sive underst-anding of the infection	Requires skilled experitise	Austria USA Columbia	[113,114,115,116]

**Table 5:** Evidence of use of modern methods of malaria detection in low and lower-middle-income countries.

Modern Method	Resource-Limited Countries	References
Malaria rapid test kit (SD Bioline RDT kit) using urine and saliva samples	Ghana	[64]
Other rapid diagnostic tests	Nigeria, Senegal, Kenya, Benin, Pakistan, Burkina Faso	[14,15,17,18,65,66,68,69]
Nested polymerase chain reaction (PCR)	Pakistan, Nigeria, Myanmar, Honduras, India	[13,16,18,23,25]
Hair qPCR	Rwanda	[29]
Other quantitative polymerase chain reaction (qPCR)	Bangladesh, Eritrea, Tanzania D.R. Congo, Sierra Leone, Cambodia	[35,36,37,38,40]
Dry LAMP system (CZC-LAMP	Zambia	[45]
Other loop-mediated isothermal amplification (LAMP),	India, Tanzania, Senegal, Ghana	[48,56,57,58,59]
Computerized/digital deep machine learning approach	Nigeria, Uganda, Bangladesh, Ethiopia, Zambia,	[59,75,77,78,79,80]
The rotating-crystal magneto-optical detection (RMOD) method	Papua New Guinea	[96]
Hemozin-based malaria diagnostic device (GazelleTM)	Honduras	[16]
Flow cytometry	Burkina Faso, India	[90,93]
Dye-coupled aptamer-captured enzyme-catalyzed assay	India	[104]
Multi-omics	India	[114]

## Data Availability

Not applicable.
